# An EEG-based analysis of the effects of different music genres on driving stress

**DOI:** 10.3389/fnhum.2025.1560920

**Published:** 2025-03-19

**Authors:** Yilun Li, Yan Li, Bangbei Tang, Qizong Yue, Bingjie Luo, Mingxin Zhu

**Affiliations:** ^1^School of Music and Dance, Henan Institute of Science and Technology, Xinxiang, China; ^2^School of Intelligent Manufacturing Engineering, Chongqing University of Arts and Sciences, Chongqing, China; ^3^Department of Physiology, Army Medical University, Chongqing, China; ^4^School of Music, Southwest University, Chongqing, China; ^5^School of Mechanical Engineering, Sichuan University of Science and Engineering, Yibin, China

**Keywords:** music intervention, emotion, brain, driving stress, driving behavior, driving safety

## Abstract

**Introduction:**

Sudden road conditions can trigger drivers’ psychological stress, increasing the risk of traffic accidents. Music, as an emotion regulation tool, effectively alleviates stress and enhances psychological health. However, the effects of different genres of music on drivers’ stress remain understudied.

**Methods:**

To address this, the present study collected 120 EEG recordings from 60 drivers in a standardized simulated driving environment and developed a classification model based on EEG signals to recognize emotions. By integrating time-frequency domain features (mean, variance, skewness, kurtosis, and power spectral density) with classification algorithms, the model accurately identified slight, moderate, and severe stress states in drivers, achieving an accuracy of 90%.

**Results:**

Furthermore, the study evaluated the intervention effects of four types of music (joyful, sorrowful, exhilarating, and gentle) on stress using EEG signals and subjective stress ratings. The results showed that gentle music had the best stress-relieving effect in both slight and severe stress states, reducing stress by 41.67% and 45%, respectively, whereas joyful music was most effective in relieving moderate stress, reducing moderate stress by 50%. In contrast, exhilarating and sorrowful music had weaker effects. Additionally, the asymmetry of frontal pole EEG signals was found to be significantly negatively correlated with stress levels.

**Discussion:**

This finding further supports the accuracy of the emotion recognition model and the potential effectiveness of the music intervention strategy. The study demonstrates that personalized music intervention strategies can help alleviate drivers’ stress, thereby improving psychological health, enhancing driving safety, and increasing driving comfort.

## 1 Introduction

The occurrence of traffic accidents has been shown to correlate with drivers’ emotional states, particularly in complex or unexpected road conditions, where driver stress is recognized as a major risk factor for accidents (Ringhand and Vollrath, 2019; Wang et al., 2022). Research indicates that emotional stress not only impairs drivers’ cognitive and reaction abilities but also leads to decision-making errors and abnormal driving behaviors, significantly increasing the risk of traffic accidents (Rastgoo et al., 2019; Guo et al., 2021; Retallack and Ostendorf, 2019). Consequently, developing effective emotional regulation strategies to alleviate driver stress has become a critical approach to enhancing traffic safety.

Emotional intervention methods primarily focus on psychological regulation and environmental optimization (Hawkins et al., 2023). Music, as a widely recognized emotional regulation tool, has been applied in various contexts to improve individuals’ emotional states (Cook et al., 2019), particularly demonstrating notable effects in alleviating negative emotions, such as stress and anxiety (Hu et al., 2020). In clinical psychology, music therapy has been widely used to treat emotional disorders such as depression, anxiety, and post-traumatic stress disorder (PTSD) (Tang et al., 2020; Lu et al., 2021; Gooding and Langston, 2019). Ueda et al. (2013) found through a meta-analysis that music therapy has a significant effect on alleviating anxiety symptoms. Carr et al. (2012) assessed the feasibility and effectiveness of group music therapy for individuals with PTSD who did not respond sufficiently to cognitive behavioral therapy (CBT). The results indicated that group music therapy had a statistically significant therapeutic effect in alleviating PTSD symptoms and co-occurring depression, and patients provided positive feedback regarding their therapeutic experience. In the treatment of sleep disorders, music therapy has demonstrated potential benefits. Wang et al. (2024) found that an intervention combining a melatonin receptor (MT2) agonist and music therapy had significant therapeutic effects on nurses with circadian rhythm sleep disorders. It improved sleep quality, alleviated anxiety and depression, and continues to hold promising prospects for clinical application. Although existing studies have confirmed that music can effectively regulate emotions, research on the specific role of music in alleviating drivers’ stress, particularly studies combining music with electroencephalographic (EEG) signal analysis, remains relatively scarce.

Electroencephalographic (EEG) signals, as important physiological indicators of brain activity, are widely utilized in emotion monitoring and recognition (Rahman et al., 2021). Due to its high temporal resolution, EEG can capture real-time emotional fluctuations, making it a valuable tool for studying emotional states (Li et al., 2022). In recent years, with advancements in emotion recognition technology, EEG has garnered attention for its potential applications in traffic safety (Halim and Rehan, 2020). Schaaff and Schultz (2009) extracted features, including the power spectrum and peak frequency of α waves from EEG signals, and employed a support vector machine (SVM) classifier to categorize emotions into happy, neutral, or unhappy categories, achieving an identification accuracy of 66.7%. Wu et al. (2017) introduced an emotion recognition method using only two frontal pole EEG channels, Fp1 and Fp2, leveraging the spatial, frequency, and asymmetry characteristics of EEG signals. The method was validated experimentally with a Gradient Boosting Decision Tree (GBDT) classifier, achieving a maximum accuracy of 76.34% and an average accuracy of 75.18% on the test dataset. Jackson et al. (2003) suggested that asymmetry in the frontal pole region may serve as a significant predictor of emotional regulation, with α wave activation in the left frontal pole region linked to the ability to suppress negative emotions. However, existing research has predominantly focused on the optimization of algorithms and the improvement of emotion recognition models, while studies integrating EEG signals with different types of music remain limited. This limitation is particularly evident in driving environments, where comprehensive investigations into the impact of music on driver emotions are still lacking.

To address these gaps, this study developed an EEG-based model to classify stress-related emotions and systematically evaluated the effects of four distinct types of music (joyful, sorrowful, exhilarating, and gentle) on drivers experiencing different levels of stress (slight, moderate, and severe). The goal of this study is to explore the application of music intervention in improving driving safety. It seeks to provide theoretical support for personalized music intervention strategies and offer data-driven insights for traffic safety management, aiming to reduce accidents and enhance driver comfort.

The main contributions of this work can be summarized as follows:

(1) This study integrates electroencephalographic (EEG) signals with music intervention, offering a novel perspective on traffic safety management by combining physiological data and personalized emotional regulation. It provides a foundation for data-driven decisions in tailoring interventions for driver safety. In the context of intelligent transportation systems, interventions targeting the driver’s emotional state can enhance traffic safety and reduce accident risks associated with emotional instability.
(2) This study classifies the driver’s stress into slight, moderate, and severe levels using EEG indicators and evaluates the effects of four distinct types of music (joyful, sorrowful, exhilarating, and gentle) on these states. This approach enables more precise and personalized interventions, providing theoretical support for future strategies tailored to the driver’s emotional state.
(3) This study evaluated the classification performance of six models across different brain regions. The results show that the frontal pole achieved the best classification performance, with the Genetic Algorithm Optimized Backpropagation Neural Network (GA-BPNN) model outperforming both the traditional Backpropagation Neural Network model and other commonly used machine learning models in terms of emotional classification accuracy.

## 2 Materials and methods

### 2.1 Screening of materials that stimulate stress and emotions

To ensure reliable elicitation of targeted emotions and consistency in experimental data, it is crucial to use pre-validated emotional stimuli, given the subjective nature of emotional arousal. This study employs a multi-channel emotion induction approach by combining video clips with visual and auditory stimuli. This combination not only enhances emotional arousal but also extends the duration of the emotional experience. The dynamic characteristics of video clips, which simulate real-life emotional events, enhance the authenticity of emotional experiences, leading to more intense subjective responses and pronounced physiological changes. Unlike previous studies that relied on pre-established emotion stimulus libraries, this study aims to construct a customized emotion-induction material library. The materials will undergo a rigorous screening process to ensure compliance with the specific requirements of the experimental design.

Emotional stimulus materials will undergo an initial screening using online platforms. The selection process will take into account factors such as participants’ cultural background, age range, educational level, and the specific objectives of the study.

Materials will be chosen based on the following criteria:

(1) The selected videos should be of short duration to minimize participant fatigue, as this could impair emotional responses. Prolonged exposure may cause exhaustion, reducing engagement and receptivity to stimuli.
(2) The selected videos must be clear and easily comprehensible to ensure effective emotional induction within a short period. Ambiguity in the stimuli may hinder the efficiency of emotional responses.
(3) The selected videos should ensure that emotion induction is singular and precise. For each stimulus material, the induced emotion must be clearly defined and free from contamination by non-targeted emotions, to minimize experimental errors.

Based on the above criteria, 10 sets of videos were selected from online platforms for further analysis by the experimenters to assess the effectiveness of the stimuli and conduct additional screening.

This study will use a five-point Likert scale to evaluate participants’ subjective emotional responses, a widely adopted method for assessing emotional reactions to various emotion regulation strategies (McCullen et al., 2023). Grounded in established psychological principles, the Likert scale quantifies individuals’ attitudes or emotions toward specific stimuli, offering a more objective and reliable method for emotional assessment (Kusmaryono et al., 2022). Emotional intensity will be measured using a five-point Likert scale, ranging from Level 1 (calm) to Level 5 (severe stress). The specific scale employed in this study is detailed in Table 1.

**TABLE 1:** Likert five-point scale.

Description	Level of stress
Calm: completely stress-free, characterized by a highly relaxed state.	1
Almost no-stress: experiencing occasional slight unease, but overall maintaining a calm state of mind.	2
Slight stress: mild discomfort, with occasional awareness of stress, but still manageable.	3
Moderate stress: a noticeable sense of unease and worry, accompanied by emotional instability that may affect cognition or behavior.	4
Severe stress: an intense sense of unease and worry, accompanied by emotional dysregulation that significantly impairs cognition and behavior.	5

Forty volunteers were recruited for the emotional stimulus material calibration experiment, and the 10 selected video clips were numbered for reference. All participants exhibited normal cognitive function, no history of depression or depressive tendencies, and the ability to articulate their emotional responses clearly. The demographic characteristics of participants in the calibration experiment are summarized in Table 2.

**TABLE 2:** Information of participants in the emotional stimuli material calibration experiment.

	Mean	Standard deviation	Median
Gender	Female 12 male 28		
Age	23.68	2.37	24
Years of driving experience	4.67	2.43	4.00

**FIGURE 1:**
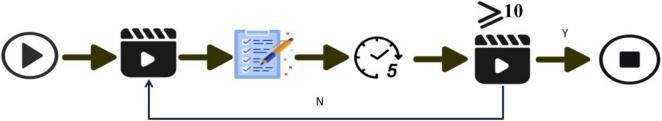
Flowchart of the emotional stimuli material calibration experiment.

The experiment was conducted in a quiet and comfortable laboratory environment, where participants were allowed to withdraw from the study at any time at their own discretion. During the experiment, participants were asked to watch the video materials sequentially and complete a five-point Likert scale evaluation for each video after viewing. After completing the evaluation, participants were given a 5 min break to stabilize their emotions before proceeding to the next video. The flowchart of the emotional stimulus material calibration experiment is shown in Figure 1.

The emotional induction effect of the stress-provoking stimuli was evaluated based on participants’ ratings on a five-point Likert scale after viewing the materials. Stress induction was considered successful if the rating exceeded Level 3 (slight stress). The success rate and rating results for the stress-provoking stimuli are summarized in Table 3.

**TABLE 3:** Calibration results of the Likert scale.

Video ID	Induction success rate	Mean score
1	57.5%	2.725
2	70%	3.2
3	55%	2.975
4	60%	2.85
5	62.5%	3.05
6	52.5%	2.575
7	87.5%	4.05
8	60%	3.075
9	67.5%	3.225
10	82.5%	3.675

As shown in Table 3, Video 7 elicited the strongest stress response, achieving a success rate of 87.5% and the highest average stress score (4.05) among all induced emotions.

Based on the analysis of the Likert scale results, the video material that most effectively induced stress was identified and selected.

### 2.2 Experimental scenario

Unlike typical work or social stressors, driver stress goes beyond mere emotional reactions to external pressures. It also encompasses the physiological and psychological burdens drivers endure during long drives or in complex traffic scenarios. Compared to other forms of stress—such as workplace or family stress—driver stress is characterized by high-stakes decision-making, intense concentration, and its inherently transient and fluctuating nature.

Due to the distinct nature of driver stress, studying its effects on emotional regulation and cognitive processes presents significant challenges. Given that this experiment involved driving under stress while recording participants’ EEG signals, real-world driving in such conditions would be both hazardous and impractical. In this study, a driving simulation experiment was employed as an alternative to real-world driving under stressful conditions. The main advantages of driving simulation experiments include enhanced safety, a highly controllable environment (e.g., temperature, lighting, and audio), and reduced costs. Numerous studies (Reyner and Horne, 1997) have demonstrated the physiological similarities between driving simulation and real-world driving. Therefore, a driving simulator was employed in this study to investigate the recognition of stress-induced emotions in drivers.

In this study, the Forza Horizon 5 software, developed on the EA platform, and the PXN-V99 driving simulator were employed to create the simulated driving environment. The simulator replicates real-world road traffic conditions experienced by drivers. It consists of three main components: the vehicle control system, the visual display system, and the audio system. The vehicle control system includes a steering wheel, gear lever, accelerator pedal, brake pedal, and clutch. The visual display system includes an LCD monitor that provides a first-person driving perspective, while the audio system delivers surround sound to enhance the realism of the driving experience.

**FIGURE 2:**
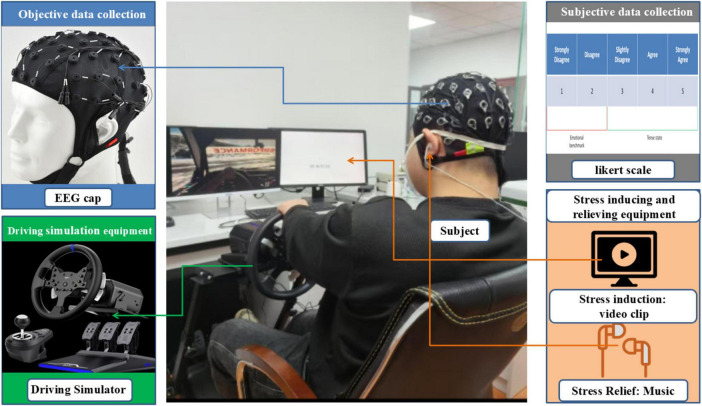
Scene setup and equipment connection diagram.

The experimental scene is shown in Figure 2. The laboratory is well-ventilated and has good lighting.

The stress-inducing video was presented using E-Prime, a human-computer interaction platform (MacWhinney et al., 2001; Richard and Charbonneau, 2009), integrated with the EEG signal acquisition module to form a synchronized data collection system. The electrode positions of the EEG cap were determined according to the international 10-10 system, with the CPz and End electrodes serving as the reference and ground, respectively. The sampling frequency was set at 500 Hz, and the electrode impedance was maintained below 5 kΩ throughout the experiment. Music was delivered through headphones connected via E-Prime.

### 2.3 Experimental procedure

A total of 60 participants, representing diverse genders and age ranges, were recruited for the formal experiment. All participants possessed a valid Chinese driver’s license and prior driving experience. Of the 60 participants, 34 were male and 26 were female. The experimental procedures were approved by the Ethics Committee of Chongqing University of Arts and Sciences (approval no. CQWL202424). Comprehensive participant information is provided in Table 4.

**TABLE 4:** Participant information.

Scene	Male	Female	Age	Years of driving experience
Expressway	34	26	20–27 (Mean = 23.32, std = 2.30)	1–9 (Mean = 4.98, std = 2.86)

To minimize potential interference with EEG signal accuracy, participants were instructed to abstain from alcohol, caffeine, nicotine, and other substances for 48 h prior to the experiment and to ensure adequate sleep. Informed consent was obtained from all participants prior to the study, detailing the experiment’s objectives and specific tasks.

At the beginning of the experiment, participants were instructed to relax for 30 s, during which their EEG signals were recorded in a calm state. Subsequently, participants were instructed to watch a stress-inducing video on the driving simulator, lasting approximately 200 s. After experiencing induced stress, participants underwent a 30 s marking period, during which the experiment assistant recorded their stress levels using the Likert five-point scale. Finally, a 30 s music regulation stage was conducted to facilitate stress recovery. The 60 participants were divided into five groups, each consisting of 12 participants. Among them, 48 participants with a stress level of three or above were subjected to music regulation using joyful, sorrowful, exhilarating, and gentle music. The remaining 12 participants, whose stress level reached level two (almost no-stress), had their EEG signals recorded without further regulation. During the regulation stage, the stress-inducing video continued to play. After the regulation was completed, the stress level was recorded again.

**FIGURE 3:**
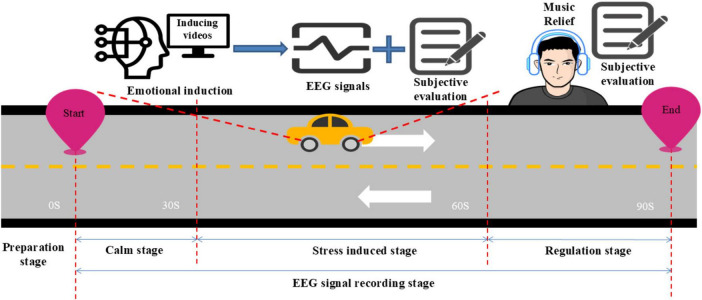
Experimental process.

The experimental process is illustrated in Figure 3.

### 2.4 Data processing and analysis

**FIGURE 4:**
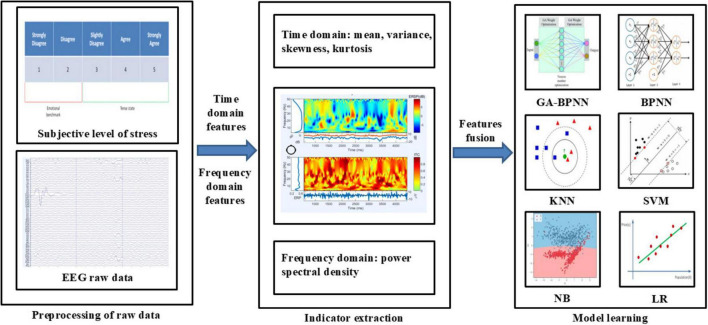
Classification algorithm flow based on electroencephalographic (EEG) signals.

The process of classifying stress-related EEG signals based on EEG data is illustrated in Figure 4 and primarily involves data preprocessing, feature extraction, and model training (Alazrai et al., 2019). Feature extraction primarily focuses on the time-domain and frequency-domain characteristics. Finally, the selected features are processed using a classification algorithm to produce the classification results.

#### 2.4.1 Data preprocessing

**FIGURE 5:**
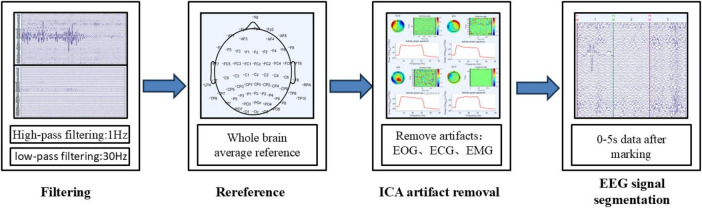
Electroencephalographic (EEG) signal preprocessing process.

The data exported from the EEG signal acquisition and analysis software are recorded directly from the scalp and often contain various types of noise and artifacts, requiring preprocessing and denoising. EEG artifacts are classified into two categories: physiological and non-physiological. Physiological artifacts are EEG signals generated by physiological activities such as blinking, eye movements, respiration, and muscle activity (Islam et al., 2016). Non-physiological artifacts primarily arise from environmental interference, with electrical noise being the most common source. Common preprocessing methods for EEG signals include filtering, re-referencing, ICA-based artifact removal, and segmentation (Pedroni et al., 2019). The preprocessing workflow for EEG signals used in this study is shown in Figure 5.

#### 2.4.2 Feature extraction

(1) Time-domain Features: Time-domain features of EEG signals play a crucial role in the feature extraction module. EEG signals are time-series signals, and the EEG time-series waveform contains a wealth of time-domain information. Extracting time-domain features of EEG signals is common in brain fatigue detection. Due to their simple calculation and ease of understanding, they are often used to analyze the state of the brain (Oh et al., 2014).

1) Mean Value: The mean value of all sampled values in the EEG signal, reflecting the overall level of the signal.


(1) $$X=\frac{1}{N}\,\sum_{i=1}^{N}x_{i}$$

2) Variance: The average of the squares of the differences between all sampled values of the EEG signal and their mean, reflecting the degree of fluctuation in the signal.


(2) $$\sigma^{2}=\frac{1}{\text{n}}\,\sum_{\text{i}=1}^{\text{n}}{\left({\text{x}}_{\text{i}}-\mu \right)}^{2}$$

3) Skewness: This feature is used to measure the asymmetry of the statistical data distribution. Skewness is defined using the third central moment and the second central moment (variance), with the calculation formula as follows:


(3) S⁢k⁢e⁢w=k3k232

(4) $$k_{3}=\frac{1}{n}\,\sum_{i=1}^{n}{\left(x_{i}-\mu \right)}^{2}$$

In the formula, *k*_3_ and *k*_2_ I represent the third central moment and the second central moment, respectively. The skewness can be positive, negative, or undefined. A positive skewness indicates a right-skewed distribution, a skewness of 0 indicates a symmetrical distribution, and a negative skewness indicates a left-skewed distribution. In general, skewness can be used to test the normality of the data.

4) Kurtosis: It is used to describe the steepness or flatness of the distribution of all values, and its definition is as follows


(5) $$\text{Kurt}=\frac{\frac{1}{\text{n}}\,\sum_{\text{i}=1}^{\text{n}}{\left({\text{x}}_{\text{i}}-\mu \right)}^{4}}{{\left(\frac{1}{\text{n}}\,\sum_{\text{i}=1}^{\text{n}}{\left({\text{x}}_{\text{i}}-\mu \right)}^{4}\right)}^{2}}-3$$

(2) Frequency-domain Features: These refer to the distribution of energy of the EEG signal at different frequencies. Changes in the signal can be obtained from changes in frequency bands, which is the main advantage of frequency-domain analysis compared to time-domain analysis.

1) Power Spectral Density (PSD): It represents the signal power per unit frequency band and is used to describe the distribution pattern of a signal as it varies with frequency within a certain region. It is a way to study signals from an energy perspective. Generally, the Fourier transform is used to convert EEG signals into frequency-domain signals within a specified frequency band. Power Spectral Density is the most common frequency-domain feature of signals.

The Welch algorithm is used to calculate the Power Spectral Density (PSD).

First, the EEG signal sequence of length N, First, the EEG signal sequence of length N, {x_i, i = 1, 2,…,N}, is divided into L segments, each with M data points. The representation of the i_th segment of data is as follows: is divided into L segments, each with M data points. The representation of the i_th segment of data is as follows:


(6) $$x_{i}\,\left(n\right)=x\,\left(n+i\,M-M\right),0≦n≦M,1≦i≦L$$

Then, a window function *w*(*n*) Tis applied to each data segment to obtain the period ogram for each segment:


(7) $$I_{i}\,\left(\omega \right)=\frac{1}{U}\,{\left|\sum_{n=0}^{M-1}x_{i}\,\left(n\right)\,w\,\left(n\right)\,e^{-j\,\omega \,n}\right|}^{2},i=1,2,\cdots ,M-1$$

(8) $$U=\frac{1}{M}\,\sum_{n\,0}^{M-1}\omega^{2}\,\left(n\right)$$

In the formula, U is a normalization factor. Under the assumption that each period ogram segment is approximately uncorrelated, the power spectral density can be expressed as follows:


(9) $$P\,S\,D\,\left(e^{j\,w}\right)=\frac{1}{L}\,\sum_{i=1}^{L}I_{i}\,\left(\omega \right)$$

Time-domain features can capture the dynamic changes and fluctuations of the signal, while frequency-domain features reveal the energy distribution of brain signals across different frequencies. During stressful emotional states, both the temporal variations and frequency components of brain signals are affected. The combined use of time-domain and frequency-domain features allows for a more comprehensive capture of emotional changes, thereby improving the accuracy of classification models.

#### 2.4.3 Establishment of a classification model based on EEG signals

The dataset in this study consists of 60 samples in a calm state (completely relaxed), 12 samples in an almost no-stress state, and 16 samples each for slight stress, moderate stress, and severe stress, totaling 120 samples.

Electroencephalographic data were collected using 31 channels: Fp1, Fp2, AF3, AF4, F7, F3, Fz, F4, F8, FC5, FC1, FC2, FC6, C5, C3, Cz, C4, C6, CP5, CP1, CP2, CP6, P7, P3, Pz, P4, P8, PO3, PO4, O1, and O2. All channels were categorized into five brain regions based on their positions: Fp (frontal pole), F (frontal), C (central), P (parietal), and O (occipital) (Wu et al., 2023).

This study extracted multiple features from five brain regions, including mean, variance, skewness, kurtosis, and power spectral density, as time-frequency domain features to serve as inputs for machine learning models. A comprehensive comparison and analysis of common machine learning classification methods and neural network prediction models was performed. The models included the traditional Backpropagation Neural Network (BPNN), the Genetic Algorithm Optimized Backpropagation Neural Network (GA-BPNN), as well as k-nearest neighbors (KNN), support vector machine (SVM), naive Bayes (NB), and logistic regression (LR). All models were implemented and simulated using MATLAB 2023a. To prevent overfitting, the leave-one-out cross-validation method was employed to evaluate model performance, with 70% of the dataset randomly selected as the training set and the remaining 30% as the test set. Classification models were constructed for each of the five brain regions: frontal pole, frontal, central, parietal, and occipital.

Organize the information as shown in Table 5.

**TABLE 5:** Overview of stress classification model based on electroencephalographic (EEG) features.

Level of stress	Number of samples	Extracted features	EEG feature channels	Algorithms used
Calm	60	Mean, variance, skewness, kurtosis, power spectral density	Frontal pole, frontal, central, parietal, occipital	GA-BPNN, BPNN, KNN, SVM, NB, LR
Almost no-stress	12			
Slight stress	16			
Moderate stress	16			
Severe stress	16			

#### 2.4.4 Model evaluation

The performance of the olfactory preference prediction model was evaluated in this study, and the model with the highest overall score was selected as the final model. Four evaluation metrics were considered: accuracy, precision, recall, and F1-score. The calculation processes for these metrics are detailed as follows.


(10) $$A\,c\,c\,u\,r\,a\,c\,y=\frac{T\,P+T\,N}{T\,P+T\,N+F\,P+F\,N}$$

(11) $$P\,r\,e\,c\,i\,s\,i\,o\,n=\frac{T\,P}{T\,P+F\,P}$$

(12) $$R\,e\,c\,a\,l\,l=\frac{T\,P}{T\,P+F\,N}$$

(13) $$F\,1-s\,c\,o\,r\,e=\frac{2\cdot P\,r\,e\,c\,i\,s\,i\,o\,n\cdot R\,e\,c\,a\,l\,l}{P\,r\,e\,c\,i\,s\,i\,o\,n+R\,e\,c\,a\,l\,l}$$

Here, TP denotes the number of true positives, TN the number of true negatives, FP the number of false positives, and FN the number of false negatives.

## 3 Result

### 3.1 Model evaluation results

**FIGURE 6:**
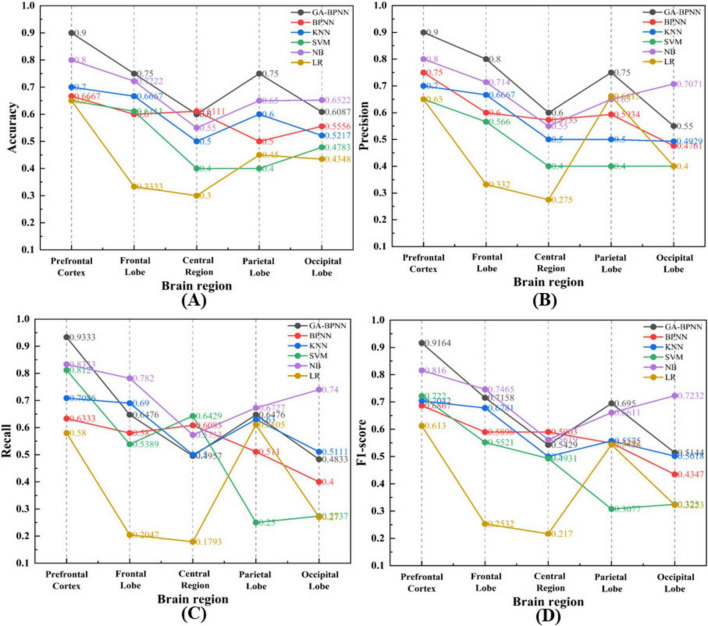
Classification results of six models across five different brain regions. **(A)** Accuracy; **(B)** Precision; **(C)** Recall; **(D)** F1-score.

Figure 6 summarizes the performance of various models across different brain regions based on accuracy, precision, recall, and F1-score. The results indicate that the GA-BPNN model outperforms the other models across all brain regions, particularly in the frontal pole, with higher classification accuracy, precision, recall, and F1-score. Therefore, the GA-BPNN model based on the frontal pole is identified as the optimal model for driver stress recognition.

**FIGURE 7:**
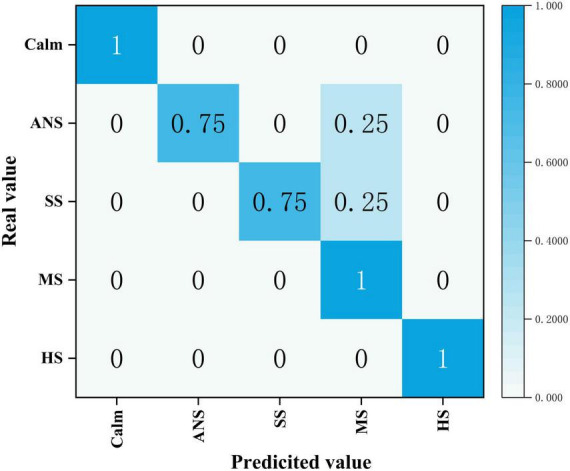
Confusion matrix of the Genetic Algorithm Optimized Backpropagation Neural Network (GA-BPNN)-based stress emotion recognition model. ANS represents “Almost No-Stress,” SS represents “Slight Stress,” MS represents “Moderate Stress,” and HS represents “High Stress.”

Figure 7 presents the confusion matrix of the GA-BPNN-based stress emotion recognition model for the frontal pole.

In this study, the input layer of the network consists of five neurons, each corresponding to one of the five features: mean, variance, skewness, kurtosis, and power spectral density. The output layer contains five neurons, each corresponding to one of the five classification labels: Calm, Almost No-Stress, Slight Stress, Moderate Stress, and High Stress. The hidden layer also consists of five neurons, and the network was trained over 36 iterations.

### 3.2 Music regulation results

**FIGURE 8:**
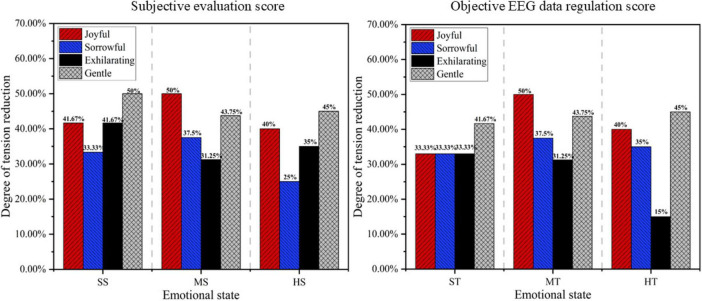
Score of subjective and objective regulation effect.

The EEG features of various types of music after regulation were fed into the pre-trained stress emotion recognition model to obtain objective EEG-based regulation scores. Additionally, the subjective evaluation scores collected during the experiment were statistically analyzed. The results were then presented as percentages, as shown in Figure 8.

As shown in Figure 8, the subjective ratings of participants on the music’s stress-regulating ability align with the objective EEG-based scores. In a slight stress state, gentle music was the most effective; in a moderate stress state, joyful music was the most effective; and in a high stress state, gentle music again yielded the best results. Overall, gentle music was identified as the most effective in stress regulation. In contrast, sorrowful and exhilarating music exhibited relatively weaker effects, particularly in high-stress states, where they failed to significantly reduce emotional fluctuations.

### 3.3 Verification of EEG asymmetric value analysis

The EEG Asymmetry Index (AI) refers to the difference in electrical activity between the left and right hemispheres of the brain and is commonly used to study brain laterality, emotions, and cognitive states (Mouri et al., 2023). It can be calculated by measuring the electrical activity in different regions of the cerebral cortex, such as α and β waves. The power subtraction method is widely recognized as a quantitative analysis technique commonly used for calculating EEG asymmetry (Bonacci et al., 2024). This method identifies functional differences between the two brain hemispheres by comparing the electrical activity, typically the power in the α or β frequency bands, between the left and right brain regions.

In this study, the frequency-domain power spectrum features of α waves were extracted, and the power law method with an exponent of two was used to calculate EEG asymmetry values for the frontal pole regions FP1 and FP2 (Harmon-Jones and Allen, 1998). Table 6 presents the EEG asymmetry values for each emotional state.

**TABLE 6:** Asymmetric electroencephalographic (EEG) values for various emotional state.

	Calm	Almost no-stress	Slight stress	Moderate stress	High stress
EEG asymmetry value	0.1340	−0.2367	−1.9866	−2.9117	−5.0518

In a calm state, the EEG asymmetry value is 0.1340, reflecting a slight positive asymmetry. This indicates that brain activity between the two hemispheres is nearly balanced, with no significant asymmetry observed (Mouri et al., 2023). In an almost no-stress state, the EEG asymmetry value is −0.2367. Although close to zero, this slight negative value suggests a minor increase in right hemisphere activity.

In a slight stress state, the EEG asymmetry value decreases to −1.9866, indicating a marked shift toward negative asymmetry. This suggests that as slight stress intensifies, right hemisphere activity increases significantly, leading to a notable enhancement in EEG asymmetry. At this stage, stress begins to exert a more pronounced effect on brain activity.

In a moderate stress state, the EEG asymmetry value further decreases to −2.9117, reflecting an intensified negative asymmetry. This indicates that with increasing stress levels, the right hemisphere’s activity becomes more dominant. Emotional and physiological changes under moderate stress begin to have a greater impact on brain activity patterns.

Finally, in a severe stress state, the EEG asymmetry value drops to −5.0518, exhibiting a significant negative shift. This demonstrates that under high stress, right hemisphere activity is strongly enhanced. At this stage, the relationship between emotional stress and EEG activity becomes highly pronounced, reflecting emotional responses such as anxiety and heightened stress (Lee et al., 2020).

Subsequently, IBM SPSS Statistics was employed to conduct a correlation analysis between stress levels and EEG asymmetry values (Cheng et al., 2020). Correlation analysis is a statistical method used to examine the relationships between two or more variables of equal importance. It enables researchers to identify relationships between variables within the data, facilitating an understanding of the degree of correlation between them. As stress level is not a strictly continuous variable but an ordinal categorical variable, the Spearman rank correlation coefficient was employed for the correlation analysis. The results are presented in Table 7.

**TABLE 7:** Verification of the correlation analysis between stress level and electroencephalographic (EEG) asymmetry value.

			Level of stress	EEG asymmetry value
Spearman rho	Level of stress	Correlation coefficient	1.000	−1.000***
		Significance (two-tailed)	0.000	0.0000
		N	5	5
	EEG asymmetry value	Correlation coefficient	1.000***	−1.000
		Significance (two-tailed)	0.000	0.0000
		N	5	5

*** Indicates significant correlation.

As shown in the table, the correlation coefficient is −1, with *P* < 0.001, indicating a significant negative correlation between stress levels and EEG asymmetry. As stress levels increase, the EEG asymmetry value decreases. The FP1 and FP2 electrodes are located in the frontal pole, indicating that this region is highly sensitive to stress-related emotions. This further validates the accuracy of stress emotion recognition models based on the frontal pole.

Subsequently, EEG asymmetry values were extracted following different types of music therapy under varying stress states, and their characteristics are summarized in Table 8.

**TABLE 8:** Asymmetric electroencephalographic (EEG) values of different music intervention under different levels of stress.

	Calm	Almost no-stress	Slight stress
Joyful	−0.2209	−0.3856	−2.1829
Sorrowful	−0.2142	−1.3467	−2.6464
Exhilarating	−0.2080	−1.6220	−2.3529
Gentle	−0.1560	−1.1064	−2.1337

**FIGURE 9:**
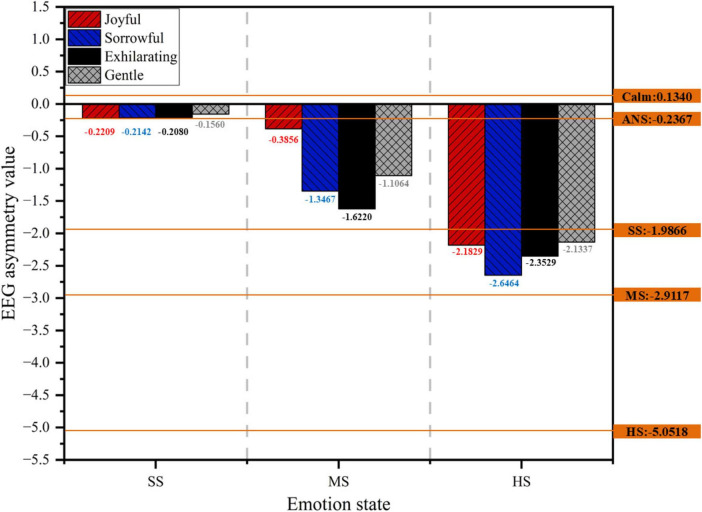
Effect diagram of stress emotion regulation based on objective electroencephalographic (EEG) data.

To facilitate observation, EEG asymmetry values under different emotional states were used as a baseline, and a bar chart was created, as shown in Figure 9. This provides a more intuitive view of the changes in EEG asymmetry values following different types of music therapy across various stress states.

Regardless of whether in a slight, moderate, or severe stress state, both gentle and joyful music are more effective in bringing EEG asymmetry values closer to those observed in calm or almost no-stress states. Both types of music effectively relax emotions, reduce stress, and promote balance in brain activity. In contrast, the effects of exhilarating and sorrowful music are less significant under stress. Overall, the EEG asymmetry values following gentle music interventions are closest to those observed in a calm or almost no-stress state. Gentle music demonstrates the most effective regulation, consistent with previous findings.

The correlation between frontal pole EEG asymmetry values and stress-related emotions further validates the accuracy of the BPNN-based frontal pole stress recognition model and the reliability of the emotion regulation assessment system.

## 4 Discuss

This study integrated EEG signals with music interventions to evaluate the effectiveness of an EEG-based stress emotion recognition model and to explore the impact of different music genres on alleviating driver stress. The results indicate that the GA-BPNN model outperforms the traditional BPNN and other commonly used machine learning models in emotion classification accuracy. Additionally, the frontal pole exhibits superior performance compared to other brain regions in classification tasks. Gentle music is the most effective in both slight and severe stress states, whereas joyful music is the most effective in moderate stress states.

### 4.1 Discussion on model prediction results

In this study, the GA-BPNN model based on the frontal pole region achieved the best performance in emotion recognition tasks, excelling in accuracy, precision, recall, and F1-score.

Among all brain regions, the frontal pole demonstrated the most outstanding performance. The frontal pole region is pivotal in emotion regulation and decision-making (Lindquist et al., 2012). As the most anterior part of the cerebral cortex, the frontal pole is commonly associated with higher cognitive functions, emotional processing, decision-making, and self-regulation. Due to its role in the recognition and regulation of emotions, its electroencephalographic signals can reliably reflect the driver’s emotional state. When confronted with sudden traffic situations, frontal pole activity exhibits significant fluctuations in response to changes in the driver’s emotional state (Souche-Le Corvec and Zhao, 2020).

Among all models, the GA-BPNN demonstrated superior classification performance. Unlike the standard BPNN model, which is often trapped in local optima during training and suffers from suboptimal parameter settings and reduced prediction accuracy, GA-BPNN leverages a genetic algorithm to optimize network parameters. This method effectively addresses these issues, significantly enhancing classification accuracy and stability, particularly in scenarios involving pronounced emotional fluctuations (Zhang et al., 2022).

Moreover, compared to traditional machine learning algorithms, GA-BPNN exhibits a clear advantage in multi-class emotion recognition tasks. Traditional machine learning methods are less effective in handling complex non-linear relationships, which are a key feature of EEG signal variations. In contrast, GA-BPNN, with its complex neural network structure, effectively captures these non-linear relationships, thereby achieving higher accuracy (Rodriguez-Bermudez and Garcia-Laencina, 2015).

### 4.2 Discussion on the intervention effect of music on emotions

Different music genres exert varying effects on emotional intervention for drivers. Gentle music demonstrated significant effectiveness in alleviating stress, reducing stress levels by 41.67% and 45% under slight and severe stress conditions, respectively. This result aligns with numerous studies on emotion regulation and music intervention. Research by Darmadi and Armiyati (2019), Teckenberg-Jansson et al. (2019) indicates that gentle, relaxing music effectively reduces autonomic nervous system excitability, alleviating negative emotional responses such as anxiety and stress. Gentle music may enhance alpha wave activity, promoting a relaxed brain state that helps drivers manage stress more effectively. Particularly in severe stress driving situations, it may regulate neural electrical activity in the brain, helping drivers restore psychological balance and reduce stress-induced attention dispersion and delayed reactions.

Joyful music demonstrated the most significant effect in moderate stress states, reducing stress levels by 50%. Joyful music often features a strong rhythm and a pleasant emotional tone, which can help drivers alleviate stress and promote positive emotional responses (Harada et al., 2017). This facilitates more effective coping with complex situations and improves cognitive control.

However, the effects of exhilarating and sorrowful music were less pronounced, particularly in severe stress states, where they failed to significantly alleviate emotional fluctuations. In severe stress situations, exhilarating music may induce excessive arousal, exacerbating stress (Lu et al., 2005), whereas sorrowful music may emotionally resonate with the driver’s stress, failing to effectively transform negative emotions (Sachs et al., 2021). The research highlights the personalized and situational dependence of music interventions, indicating that their emotional regulation effects are dynamic and closely tied to the music’s emotional content, the driver’s emotional state, and the specific context. Therefore, it is essential to select the most appropriate type of music based on an individual’s emotional sensitivity, preferences, and psychological state.

This study evaluated the effects of different music genres on participants under varying stress states from both subjective and objective perspectives, thereby enhancing the credibility and scientific rigor of its findings. Subjective data (Likert scale) reflects individuals’ internal experiences, whereas objective data (EEG signals) provides external validation. The mutual corroboration of these two data types enhances the robustness of the study’s conclusions.

### 4.3 The relationship between EEG asymmetry and emotions

The study further analyzed the relationship between EEG signals and drivers’ emotional states, finding a significant negative correlation between the EEG asymmetry value of alpha waves (FP1-FP2) in the frontal pole and the driver’s level of stress. This conclusion is consistent with the findings of Zhao et al. (2018). EEG asymmetry typically reflects differences in activity between the brain’s left and right hemispheres. In emotion regulation, left hemisphere activity is generally associated with positive emotions and relaxed states, whereas the right hemisphere is linked to negative emotions such as stress and anxiety, with more pronounced activation observed in the right hemisphere during negative emotional states (Harmon-Jones and Allen, 1998). As emotions gradually stabilize or shift toward a more positive state, left hemisphere activity increases, and the EEG asymmetry value approaches a balanced state. Gentle music can serve as an effective tool for emotional recovery by facilitating the rapid regulation of brain activity and the restoration of emotional balance.

### 4.4 Limitations and future jobs

Although this study produced several meaningful findings, it has certain limitations. First, the sample size is relatively small, which may limit the generalizability and robustness of the results. However, it is important to note that the current study represents a preliminary investigation in this field. Second, the study considered only four music genres and three stress states, without exploring the interactions between a broader range of music genres and emotional states. Moreover, although EEG provides a direct measurement of brain activity, emotion is inherently complex and multidimensional. Combining other physiological indicators (such as heart rate variability) with behavioral data may be necessary to comprehensively assess the effects of music and explore the predictive power of different signal combinations. Finally, future work will focus on exploring improved model frameworks and optimization methods to enhance prediction accuracy.

## 5 Conclusion

In summary, this study developed a driver stress recognition model capable of accurately identifying specific stress levels by utilizing both time-domain features (mean, variance, skewness, kurtosis) and frequency-domain features (power spectral density) derived from drivers’ EEG signals. The model was applied to evaluate the intervention effects of four music genres (joyful, sorrowful, exhilarating, and gentle) on drivers under varying stress levels (slight, moderate, and severe), proposing a novel music intervention strategy that integrates EEG signal features with personalized music to regulate emotional states and alleviate stress. Building on this strategy, future systems could monitor drivers’ emotional fluctuations in real time and play tailored music, harnessing the therapeutic potential of music to maintain positive emotional states, thereby improving psychological well-being, driving safety, and overall comfort. Furthermore, the findings of this study could inform the development of portable emotion regulation devices, enabling seamless integration of real-time emotion recognition and music intervention technologies, paving the way for innovative applications in intelligent transportation, psychological health, and human-computer interaction, while offering new solutions to enhance music-based interventions and improve quality of life.

## Data Availability

The original contributions presented in this study are included in this article/supplementary material, further inquiries can be directed to the corresponding authors.
